# Clinical Persistence of Chlamydia trachomatis Sexually Transmitted Strains Involves Novel Mutations in the Functional αββα Tetramer of the Tryptophan Synthase Operon

**DOI:** 10.1128/mBio.01464-19

**Published:** 2019-07-16

**Authors:** Naraporn Somboonna, Noa Ziklo, Thomas E. Ferrin, Jung Hyuk Suh, Deborah Dean

**Affiliations:** a Center for Immunobiology and Vaccine Development, University of California San Francisco Benioff Children’s Hospital Oakland Research Institute, Oakland, California, USA; b Department of Pharmaceutical Chemistry, University of California, San Francisco, California, USA; c Department of Bioengineering, University of California Berkeley and University of California San Francisco Joint Graduate Group, Berkeley and San Francisco, California, USA; d Department of Medicine and Pediatrics, University of California, San Francisco, California, USA; Sequella, Inc.; Louisiana State University School of Medicine; SUNY Downstate Medical Center

**Keywords:** *Chlamydia trachomatis*, indole, interferon gamma, sexually transmitted infections, *trpA*, tryptophan synthesis

## Abstract

Clinical persistence of Chlamydia trachomatis (*Ct*) sexually transmitted infections (STIs) is a major public health concern. *In vitro* persistence is known to develop through interferon gamma (IFN-γ) induction of indoleamine 2,3-dioxygenase (IDO), which catabolizes tryptophan, an essential amino acid for *Ct* replication. The organism can recover from persistence by synthesizing tryptophan from indole, a substrate for the enzyme tryptophan synthase. The majority of *Ct* strains, except for reference strain B/TW-5/OT, contain an operon comprised of α and β subunits that encode TrpA and TrpB, respectively, and form a functional αββα tetramer. However, *trpA* mutations in ocular *Ct* strains, which are responsible for the blinding eye disease known as trachoma, abrogate tryptophan synthesis from indole. We examined serial urogenital samples from a woman who had recurrent *Ct* infections over 4 years despite antibiotic treatment. The *Ct* isolates from each infection episode were genome sequenced and analyzed for phenotypic, structural, and functional characteristics. All isolates contained identical mutations in *trpA* and developed aberrant bodies within intracellular inclusions, visualized by transmission electron microscopy, even when supplemented with indole following IFN-γ treatment. Each isolate displayed an altered αββα structure, could not synthesize tryptophan from indole, and had significantly lower *trpBA* expression but higher intracellular tryptophan levels compared with those of reference *Ct* strain F/IC-Cal3. Our data indicate that emergent mutations in the tryptophan operon, which were previously thought to be restricted only to ocular *Ct* strains, likely resulted in *in vivo* persistence in the described patient and represents a novel host-pathogen adaptive strategy for survival.

## Abstract (precis)

Chlamydia trachomatis (*Ct*) is the most common sexually transmitted bacterium with more than 131 million cases occurring annually worldwide. *Ct* infections are often asymptomatic, persisting for many years despite treatment. *In vitro* recovery from persistence occurs when indole is utilized by the organism’s tryptophan synthase to synthesize tryptophan, an essential amino acid for replication. Ocular but not urogenital *Ct* strains contain mutations in the synthase that abrogate tryptophan synthesis. Here, we discovered that the genomes of serial isolates from a woman with recurrent, treated *Ct* STIs over many years were identical with a novel synthase mutation. This likely allowed long-term *in vivo* persistence where active infection resumed only when tryptophan became available. Our findings indicate an emerging adaptive host-pathogen evolutionary strategy for survival in the urogenital tract that will prompt the field to further explore chlamydial persistence, evaluate the genetics of mutant *Ct* strains and fitness within the host, and their implications for disease pathogenesis.

## INTRODUCTION

Sexually transmitted Chlamydia trachomatis (*Ct*) infections are highly prevalent with estimates of more than 131 million global cases occurring annually (1). They represent a major public health concern due to increasing rates worldwide and severe complications of the female reproductive tract that include tubal factor infertility, ectopic pregnancy, and chronic pelvic pain (2–4). *Ct* can also cause trachoma, a chronic ocular infection that can lead to blindness (5, 6).

Sexually transmitted infections (STIs) with *Ct* can persist for long periods of time (7–9). Most uncomplicated urogenital infections can be treated with a single dose of azithromycin with an efficacy of ∼90% (10, 11). Paradoxically, treatment can also lead to a subsequent increase in the rates of urogenital infections due to interference with the development of effective immune defense against reinfection (12–14), known as the arrested immunity hypothesis (15). In *in vitro* experiments, antibiotic treatment with penicillin, rifampin, or azithromycin results in development of a persistent, nonreplicative aberrant form of the organism that can recover on removal of the antibiotic (16, 17). This may be one explanation for the recent decrease in the clinical efficacy of azithromycin (18–20).

Other factors can also cause *in vitro* persistence. In studies of human epithelial cells, interferon gamma (IFN-γ) induces the indoleamine 2,3-dioxygenase (IDO) enzyme that degrades tryptophan, an essential amino acid (aa) for *Ct* replication (21), and thereby inhibits *Ct* growth, forcing the organism into a persistent state. Growth resumes when tryptophan is added back to the medium (22, 23).

The genome of this obligate intracellular human pathogen has been shown to contain a tryptophan synthase operon comprised of genes encoding the tryptophan repressor (TrpR) and tryptophan synthase α and β subunits (TrpA and TrpB, respectively) (24). Mutations in *trpA* were previously identified among trachoma strains A, Ba, and C, suggesting that this gene is involved in organotropism (25). The mutations resulted in a frameshift that was subsequently shown *in vitro* to render the enzyme nonfunctional (24, 25).

Previously, the urogenital *Ct* TrpA was found to be incapable of synthesizing indole from indole-3-glycerol phosphate (IGP) (24). However, TrpB has been shown to catalyze tryptophan production from indole, which requires a full-length TrpA protein to form a proper αββα tetramer with a tunnel for substrate binding and full synthase activity (26). Hence, only *Ct* strains with an intact operon can be rescued in *in vitro* experiments when indole is added back to the medium (25, 27, 28). However, it is conceivable that the organism could undergo selective pressure in an IFN-γ-rich environment, such as the urogenital tract, that may promote advantageous mutations close to the active ligand-binding site of indole and/or α and β subunit interaction sites. This could affect the structure and function of the tetramer, resulting in establishment of persistent infections *in vivo* for long-term *Ct* survival, although this has not been shown in human populations.

Here, we examined serial isolates of *Ct* urogenital F strains from a woman who had had recurrent *Ct* STIs over a number of years despite antibiotic treatment. Our data indicate that mutations in the tryptophan synthase operon of these urogenital strains altered tetramer function and likely resulted in the *in vivo* persistence of the isolate in the patient described, indicating a selective adaptive host-pathogen evolutionary strategy for survival.

## RESULTS

### C. trachomatis clinical F strains I to IV contain a frameshift deletion mutation causing *trpA* elongation and cluster as a phylogenetic subbranch of urogenital strains.

**FIG 1:**
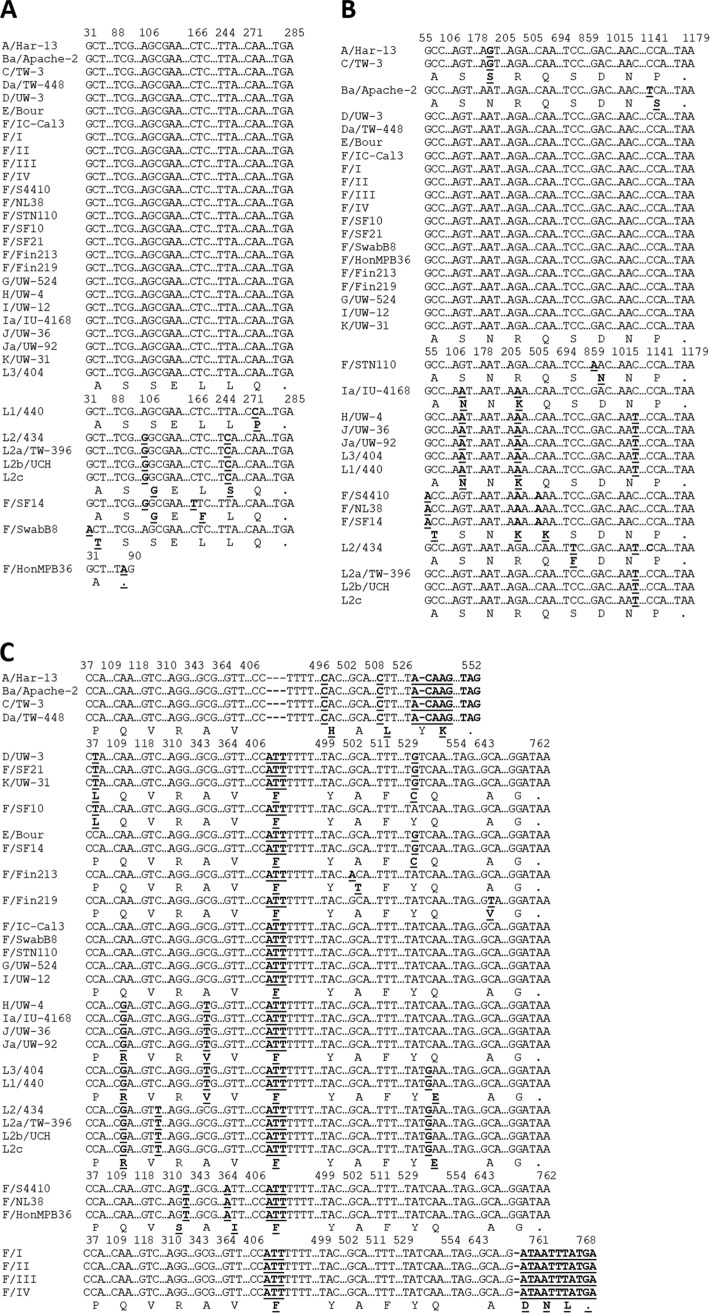
C. trachomatis clinical strains F I to IV (F I-IV) contain a *trpA* frameshift causing TrpA elongation. (A to C) Partial nucleotide sequences showing *trpR* (A), *trpB* (B), and *trpA* (C) polymorphisms of the four serial clinical strains F I-IV compared to 20 C. trachomatis (*Ct*) reference strains (A/HAR13, Ba/Apache2, C/TW3, D/UW3, Da/TW448, E/Bour, F/ICCal3, G/UW57, H/UW4, I/UW12, Ia/IU4168, J/UW36, Ja/UW92, K/UW31, L1/440, L_2_/434, L_2_a/TW396, L_2_b/UCH-1/proctitis, L_2_c, and L_3_/404) including novel clinical F strains from the San Francisco Bay Area (*n* = 7) and all F strains previously sequenced and available from public databases (*n* = 59). A period in the sequence denotes homologous sequences that are not shown. Dashes denote nucleotide deletions at positions A408, T409, T410, and T528 for *trpA* of ocular strains and at position 758 for *trpA* of clinical strains F I-IV. Bold nucleotide letters denote substitution mutations, while bold amino acid letters denote nonsynonymous amino acid substitutions. Strains with homologous sequences are not shown: F/SotonF1-F4, F/Soton18-137, F/R4663-28312, F/STN15-22, F/UK35155-770010, F/SW4-5, F/SWFP, F/S1470-3948, F/NI1, F/NL30-36, F/Aus20, F/C55, F/It686-688, F/Sou9-100, F/Swab5, F/SwabB5, F/Fin106-219, and F/SF7-19. These strains had genes that were similar to the *trpR* gene of A/HAR13-L3/404, the *trpB* gene of D/UW-3-K/UW-31, and the *trpA* gene of F/IC-Cal3-I/UW-12.

Clinical strains were isolated from a female who had recurring infections over a period of 4 years denoted F I to IV (F I-IV). The four strains had identical genomes (GenBank accession no. SRA051574.1 [29]), suggesting persistent infection with the same strain. *ompA*, a standard gene used for *Ct* genotyping, differed by a single nucleotide polymorphism (SNP) from reference strain F/IC-Cal3, encoding a synonymous aa mutation. *trpR* and *trpB* sequences were identical to those of the reference strain (Fig. 1A and B); *trpA* sequences contained a deletion at nucleotide (nt) 758, compared with F/IC-Cal3, causing a nonsynonymous aa mutation, G253D, and elongation of TrpA by two aas (254N 255L) (Fig. 1C). These data were compared to the 20 known *Ct* reference strains, and none had this mutation.

Of seven novel San Francisco clinical F strains, F/SF10 was identical to D/UW3 and K/UW31 at the N terminus and to E/Bour at the C terminus (Fig. 1C). F/SF14 was similar to E/Bour, while F/SF21 was identical to K/UW31 and D/UW3 (Fig. 1C). Of the 59 publicly available F sequences, F/HonMPB36 shared an identical *trpA* sequence with F/NL38 and F/S4410, while *trpR* was truncated after 90 bp (Fig. 1A to C).

**FIG 2:**
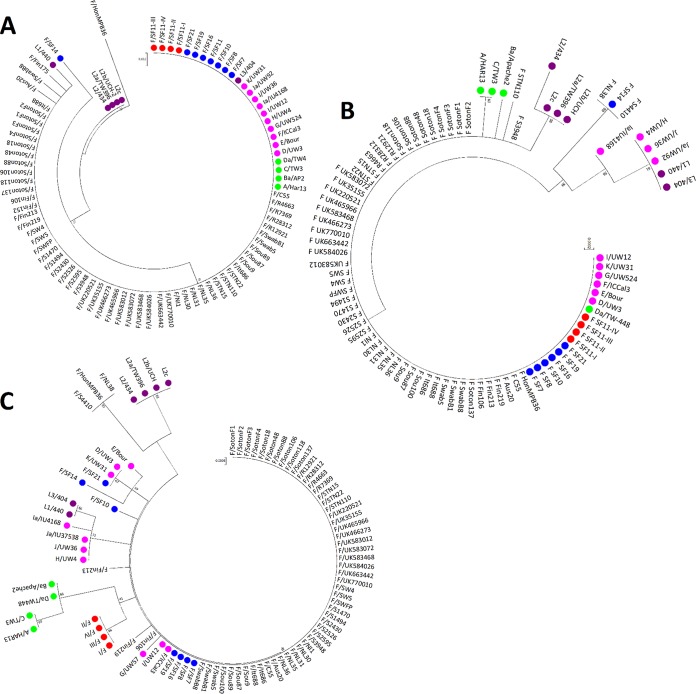
Phylogeny of *trpRBA* for 20 *Ct* reference strains, clinical F I-IV strains and other publicly available C. trachomatis F strains clustered F I-IV as a subbranch of reference strain F/IC-Cal3. (A to C) *trpR* (A), *trpB* (B), and *trpA* (C) maximum likelihood trees were constructed using Tamura-3-parameters method with 10,000 bootstrap replicates of the F I-IV strains, seven novel clinical F strains collected from San Francisco Bay Area clinics, 59 *Ct* clinical F strains available from public databases, and the 20 reference strains of *Ct*. B/UW-3 contained no *trpRBA* operon and therefore was not included. Scale bar at the top and at each branch length correspond to sequence divergence scale and time. The numbers at branches indicate bootstrap score. Red dots, F I-IV strains; blue dots, San Francisco F strains; magenta dots, reference *Ct* strains; green dots, ocular *Ct* strains; maroon dots, LGV strains.

*trpA* phylogeny clustered F I-IV as a subbranch of F/IC-Cal3 (Fig. 2). The tree differentiated ocular trachoma strains (A, Ba, C, and Da) from noninvasive urogenital (D-K) and invasive lymphogranuloma venereum (L_1_-L_3_) strains, which is similar to previous phylogenetic analyses of this gene. TrpA phylogeny was similar to *trpA* (see Fig. S1 in the supplemental material).

10.1128/mBio.01464-19.2 FIG S1 Phylogenetic trees of TrpRBA for C. trachomatis reference and clinical F strains. (A to C) TrpR (A), TrpB (B), and TrpA (C) maximum likelihood trees were constructed using JTT matrix-based model method with 500 bootstrap replicates of the F I-IV strains, seven novel clinical F strains collected from San Francisco Bay Area clinics, 59 *Ct* clinical F strains available from public databases, and the 20 reference strains of *Ct*. Scale bar at the top and at each branch length correspond to sequence divergence scale and time. Numbers at branches indicate bootstrap score. Red dots, F I-IV strains; blue dots, San Francisco F strains; magenta dots, reference *Ct* strains; green dots, ocular *Ct* strains; maroon dots, LGV strains. Download FIG S1, TIF file, 1.1 MB. Copyright © 2019 Somboonna et al. 2019 Somboonna et al. This content is distributed under the terms of the Creative Commons Attribution 4.0 International license.

### Comparative structure analyses of TrpB and TrpA for clinical strains F I-IV reveals mutations within the functional αββα tetrad complex.

**FIG 3:**
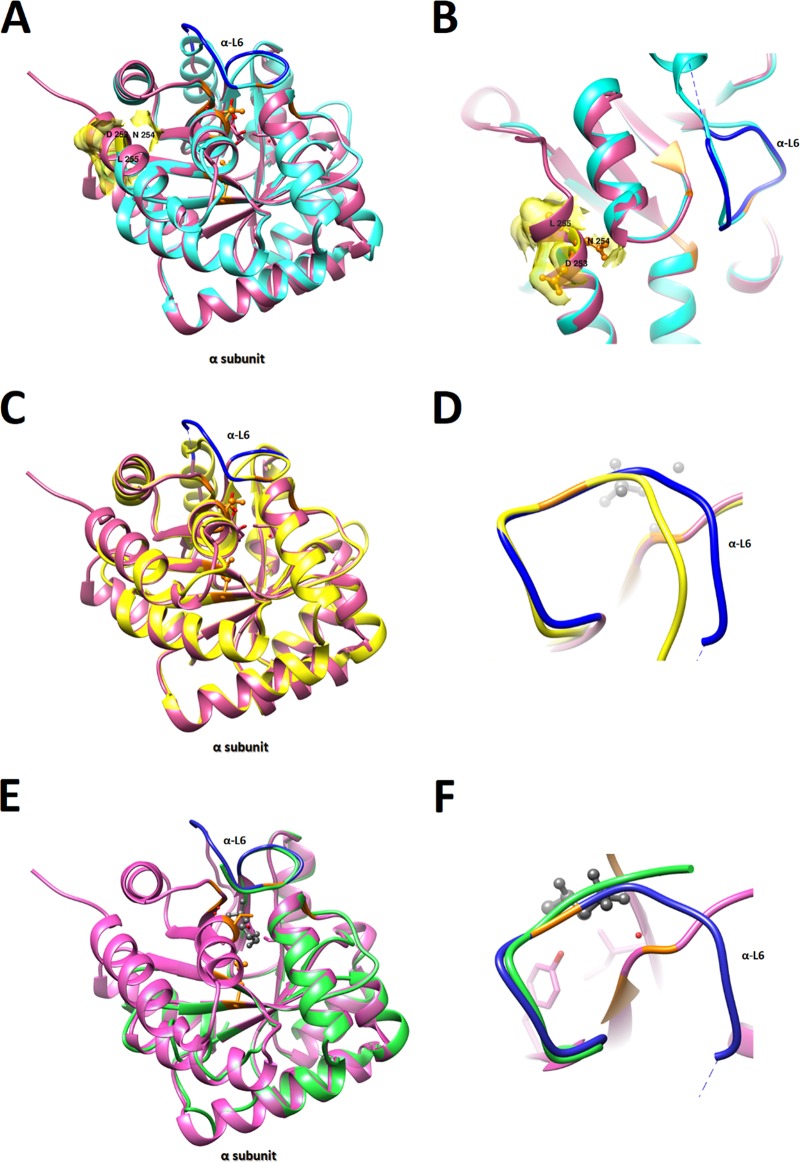
TrpA 3D predicted structures of clinical and reference C. trachomatis strains. (A to F) TrpA structures of *Ct* clinical strains F I-IV (cyan) (A), elongation of clinical strain F α254N and α255L aa surface area (yellow) and α-loop L6 (α-L6) of template 5tch (blue) and clinical strain F (cyan) (B), *Ct* urogenital reference strain F/IC-Cal3 (yellow) (C), α-L6 of template 5tch (blue) and F/IC-Cal3 (yellow) (D), *Ct* ocular reference strain A/HAR13 (green) (E), and α-L6 of template 5tch (blue) and A/HAR13 (green, truncated) (F). All structures were constructed using the template from the published crystal structures of the reconstructed putative last bacterial common ancestor (LBCA) tryptophan synthase 5ey5 (magenta) using MODELLER with visualization construction using Chimera (see Materials and Methods). Residues involved in both catalytic and subunit interaction are shown in orange. Dashed lines represent the tunnel and substrate binding site at the α subunit.

F I-IV TrpA was elongated by two aas, 254N and 255L, to a total of 255 aas. Since there are no crystal structures of the *Ct* tryptophan operon proteins, we used MODELLER (30) to model the α and β subunits based on homologies to known crystal structure templates. The TrpA structure (Fig. 3A, cyan) had >95% correct folds and a GA341 MODELLER score of 1.0, indicating a highly reliable modeling structure (31), and aligned well with templates last bacterial common ancestor (LBCA) 5ey5 (32), which had the highest identity to the clinical F TrpA of 31.76% (Fig. 3A, magenta), Mycobacterium tuberculosis 5tch (28.63% identity), and Salmonella enterica serotype Typhimurium 1qop (25.1% identity). The elongation was located at the end of the protein (Fig. 3A and B, yellow surface of ribbons), rather than at the active site. F/IC-Cal3 TrpA, which is known to be functional, had good homology to the 5ey5 structure (32.02% identity) (Fig. 3C and D, yellow), while A/HAR13 had 34.43% similarity for the 183 aas but only 23.07% similarity to the entire 5ey5 protein (Fig. 3E and F; green).

**FIG 4:**
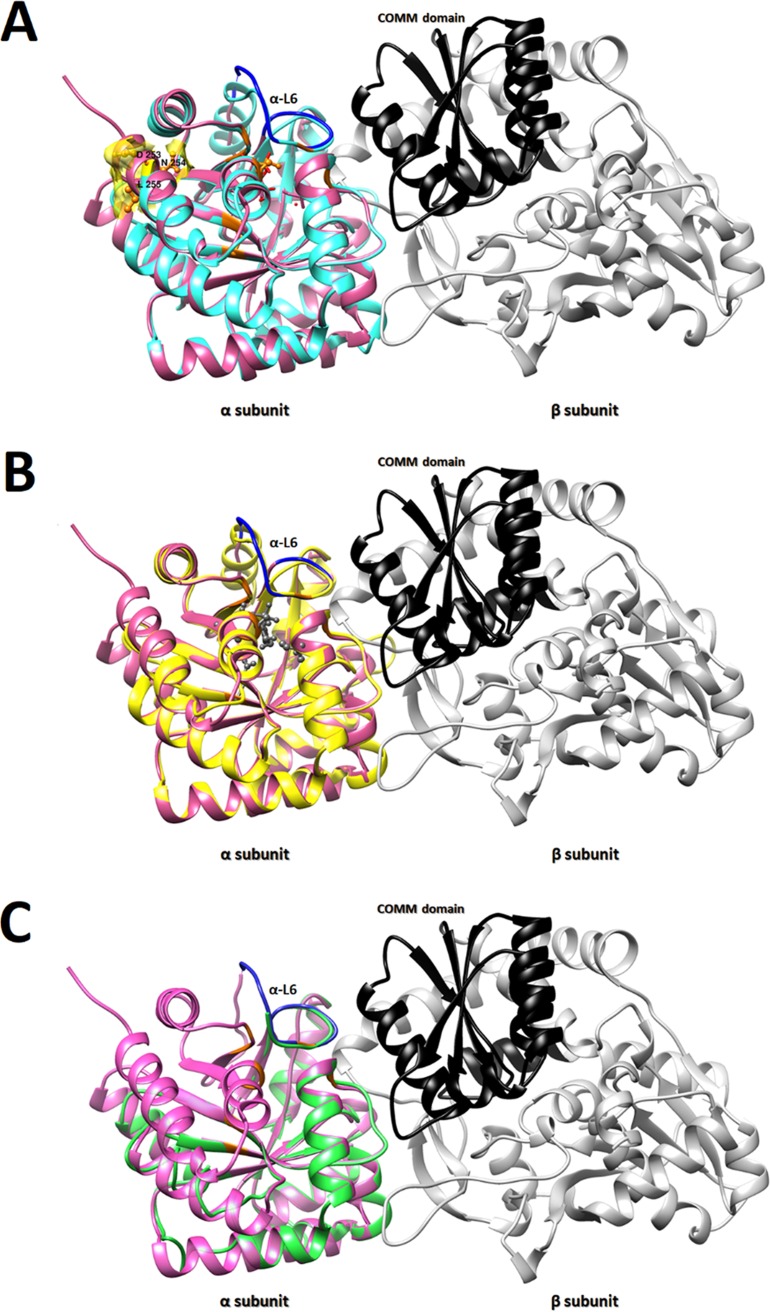
Comparative structure analyses of TrpB and TrpA for clinical strains F I-IV reveals mutations within the functional αββα tetrad complex. (A to C) TrpA-TrpB dimer structures of *Ct* clinical strain F (cyan) (A), *Ct* urogenital reference strain F/IC-Cal3 (yellow) (B), and *Ct* ocular reference strain A/HAR13 (green) (C). The TrpB structure is shown with the β-COMM domain in black. All TrpA and TrpB structures were constructed using the template from the published crystal structures of the reconstructed putative last bacterial common ancestor (LBCA) tryptophan synthase 5ey5 (magenta) using MODELLER with visualization construction using Chimera (see Materials and Methods). α-L6 residues are shown in blue, and residues involved in both catalytic and subunit interaction are shown in orange.

Dimer subunits α and β have either an open or closed conformation. Closing of the α subunit is associated with α-loop L6 (α-L6) mobility that requires residues α176 to α196 (Fig. 3B and D), which, together with the β-COMM domain (Fig. 4A to C, bold black), are critical for the closed conformation and formation of the tunnel that traps indole (33). A/HAR13 has a predicted three-dimensional (3D) model that does not have a complete α-L6 (Fig. 3E and F, green) due to truncation at residue 183 in proximity to the β-COMM domain (Fig. 4C, black) and no proximal alpha helix (Fig. 3F, arrow). Dimers for clinical F and F/IC-Cal3 had almost identical sequences and exhibited comparable structures with >95% correct folds (Fig. 4A and B) and a GA341 MODELLER score of 1.0.

**FIG 5:**
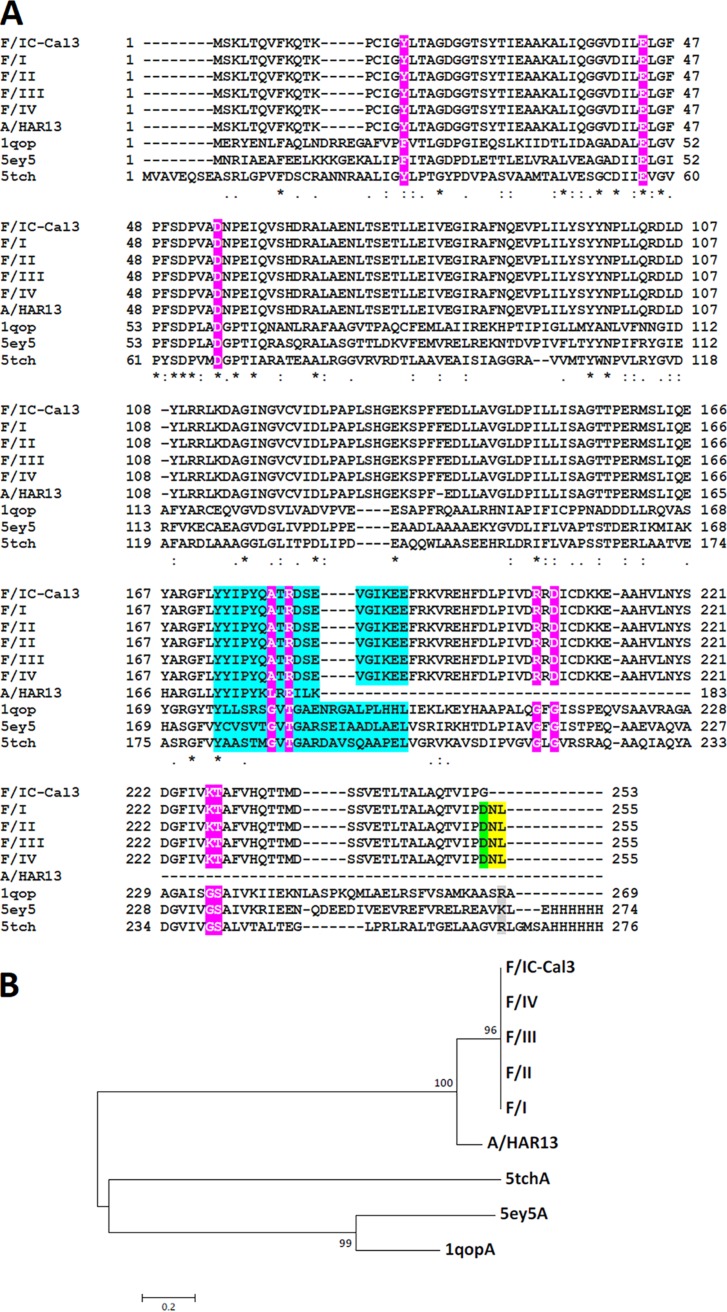
TrpA amino acid alignment and phylogeny of C. trachomatis clinical strains F I-IV, A/HAR13, F/IC-Cal3, and Protein Data Bank (PDB) templates. (A) For 3D model reconstructions, amino acid alignments of *Ct* clinical strains F/I-IV, F/IC-Cal3, and A/HAR13 with the PDB templates of LBCA 5ey5, M. tuberculosis 5tch, and *S.* Typhimirium 1qopA was performed using Clustal Omega (see Materials and Methods). Residues involved in both catalytic and subunit interaction (magenta), α-L6 residues α176-196 according to the 5tch sequence (cyan), clinical strain F mutation G253D and .254N and .255L aa elongation (yellow), gray, basic amino acids arginine and lysine in template sequences (gray), and acidic amino acid aspartic acid in clinical strains (green) are indicated. (B) Phylogenetic tree of *Ct* strains and PDB aa templates using neighbor joining with Jones-Taylor-Thornton (JTT) substitution method with 10,000 bootstrap replications using MEGA 7 (see Materials and Methods).

The TrpA aa alignment of F/IC-Cal3, A/HAR13, and F I-IV along with templates 5ey5, 5tch, and 1qop are shown in Fig. 5A. Highlighted in magenta are residues located around the tunnel comprised of Phe^22^, Glu^49^, Asp^60^, Thr^183^, Gly^211^, Gly^213^, Gly^234^, and Ser^235^ that are based on the 1qop sequence. α-L6 residues α176-196 are in cyan. The clinical F mutation at α253 is highlighted in green, and protein elongation in yellow. Figure 5B shows the TrpA phylogenetic tree.

### Recoverable infectivity of C. trachomatis clinical strains F I-IV was significantly lower than for F/IC-Cal3 following tryptophan starvation and rescue.

**FIG 6:**
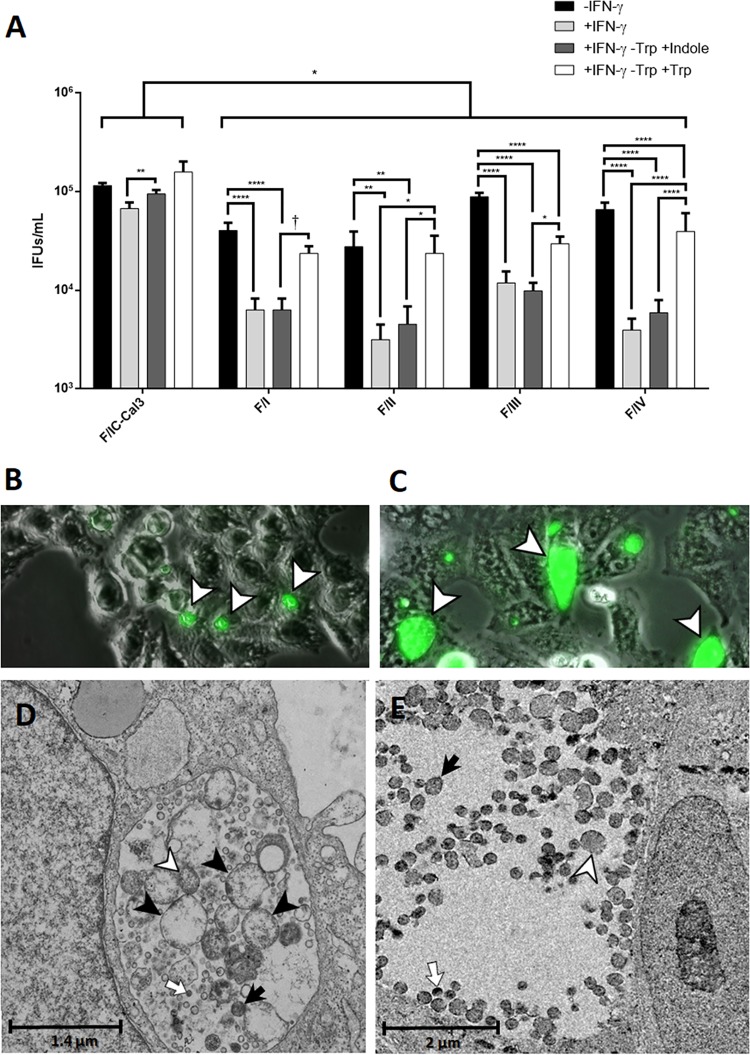
Recoverable infectivity of C. trachomatis clinical strains F I-IV was significantly lower than for F/IC-Cal3 following tryptophan starvation and rescue. Infected cells were incubated for 48 h in the presence of either complete DMEM medium (–IFN-γ), tryptophan-free DMEM medium treated with 5 ng/ml human IFN-γ (+IFN-γ), tryptophan-free DMEM medium treated with 5 ng/ml human IFN-γ supplemented with 50 μM Indole (+IFN-γ –Trp +Indole) or 10 mg/liter tryptophan (+IFN-γ –Trp +Trp). (A) Comparison between reference strain F/IC-Cal3 and clinical strains F I-IV. Mean recoverable IFUs/ml plus standard deviations (SD) (error bars) from three independent experiments. **†**, *P = *0.0597; *, *P < *0.05; **, *P < *0.01; ****, *P < *0.0001. (B) Inclusion morphology of F/IC-Cal3 under IFN-γ and indole treatment (+IFN-γ –Trp +Indole). Inclusions were stained with a FITC-conjugated *Ct*-specific LPS MAb (see Materials and Methods). White arrowheads indicate *Ct* inclusions. (C) Clinical strain F II under IFN-γ and indole treatment (+IFN-γ –Trp +Indole); inclusions were stained with a *Ct*-specific LPS-MAb at 48 hpi. The morphology of F I, III, and IV were similar to that of F II (data not shown). Arrowheads indicate *Ct* inclusions. (D) TEM of clinical strain F II and (E) reference strain F/IC-Cal3 under IFN-γ and indole treatment (+IFN-γ –Trp +Indole) at 48 hpi. White arrow, Elementary Body; Black arrow, Intermediate Body; white arrowhead, Reticulate Body; black arrowhead, Aberrant Body.

To assess whether F I-IV can synthesize tryptophan from indole despite mutations in the α subunits of the tryptophan operon, *in vitro* tryptophan starvation and indole rescue assays were performed, comparing F I-IV and F/IC-Cal3 (see Materials and Methods). F I-IV were not able to recover from tryptophan starvation using indole (Fig. 6A) at 48 h postinfection (hpi); these strains (+IFN-γ –Trp +indole) had significantly fewer inclusion-forming units per milliliter (IFUs/ml) compared to the same strains receiving media without IFN-γ (–IFN-γ) (*P < *0.01). Furthermore, when tryptophan was added (–IFN-γ –Trp +Trp), recoverable IFUs/ml were significantly higher than the starved strains supplemented with indole (+IFN-γ –Trp +indole) (*P < *0.05). F/IC-Cal3 had an overall higher growth rate in comparison to F I-IV for every condition (*P < *0.05). The addition of indole to the tryptophan-depleted media (+IFN-γ –Trp +indole) was sufficient to yield significantly higher IFUs/ml compared to IFN-γ-treated F/IC-Cal3 (+IFN-γ) (*P < *0.01).

To further evaluate the lack of recovery for F I-IV, inclusion morphology was examined under IFN-γ and indole treatment (+IFN-γ –Trp +Indole) at 48 hpi. Clinical F strain inclusions were considerably smaller (∼100 to 200 μm^2^; Fig. 6C) than those formed by F/IC-Cal3 (∼500 to 600 μm^2^; Fig. 6B). In addition, under the same treatment conditions, transmission electron microscopy (TEM) images showed that clinical strain F II contained inclusion bodies with numerous aberrant bodies compared with F/IC-Cal3 at 48 hpi (Fig. 6D and E). The morphology and TEM findings were similar for F I, III, and IV (data not shown).

### *trpBA* mRNA expression for clinical strains F I-IV was significantly reduced compared to F/IC-Cal3 under tryptophan depletion and rescue.

**FIG 7:**
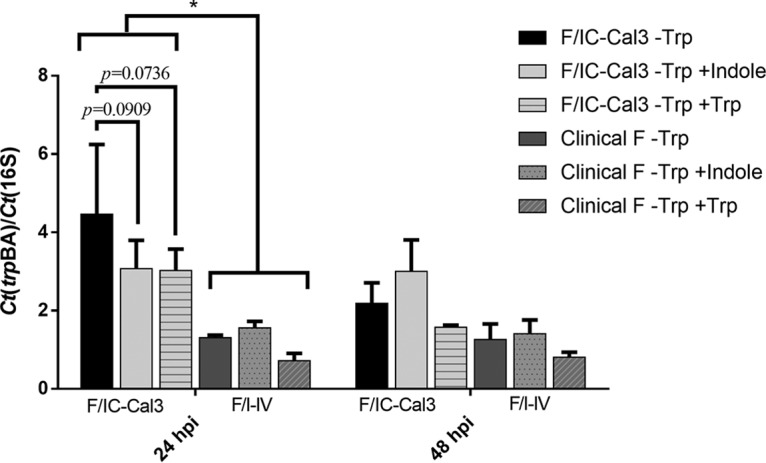
*trpBA* mRNA expression levels for clinical strains F I-IV were significantly reduced compared to F/IC-Cal3 under tryptophan depletion and rescue. At 48 h before infection, HeLa cells were treated with 5 ng/ml of human IFN-γ for 24 h in tryptophan-free media. Infected cells were incubated in the presence of either tryptophan-free DMEM (–Trp), tryptophan-free DMEM supplemented with 100 μM indole (–Trp +Indole), or tryptophan-free DMEM supplemented with 10 mg/liter tryptophan (–Trp +Trp). Infected cells were harvested at indicated times. RNA was reversed transcribed to cDNA, and expression levels were normalized to the *Ct* 16S rRNA and presented as means plus SD from three independent experiments. Data are shown for F I, although F II-IV had similar mRNA transcription patterns. There were no statistically significant differences at 48 hpi. *, *P < *0.05.

To verify F/IC-Cal3 tryptophan operon activity compared to F I-IV, *trpBA* mRNA expression levels were measured at 24 and 48 hpi under the conditions of tryptophan starvation and rescue with either indole or tryptophan (Fig. 7). At 24 hpi, during tryptophan starvation (–Trp) and rescue with indole (–Trp +Indole) or tryptophan (–Trp +Trp), F/IC-Cal3 had significantly higher expression levels of *trpBA* compared to F I-IV (Fig. 7). Additionally, there was a trend in upregulation of F/IC-Cal3 *trpBA* expression when incubated in tryptophan-depleted media (–Trp) compared with indole (–Trp +Indole; *P = *0.0909) and tryptophan supplementation (–Trp +Trp; *P = *0.0736), indicating activity of the tetrad during tryptophan starvation. *trpBA* levels were also measured at 12, 36, and 60 hpi; F/IC-Cal3 levels were upregulated starting at 12 hpi, whereas F I-IV *trpBA* levels were relatively similar throughout development with 4.33-, 2.25-, and 3.16-fold lower levels than F/IC-Cal3, respectively (data not shown). *trpR* expression levels showed a similar trend, in which indole supplementation after tryptophan starvation was not sufficient to downregulate *trpR* in clinical F strain (data not shown).

### *In vitro* intracellular tryptophan concentrations vary significantly for reference strain F/IC-Cal3 compared with clinical strains F I-IV.

**FIG 8:**
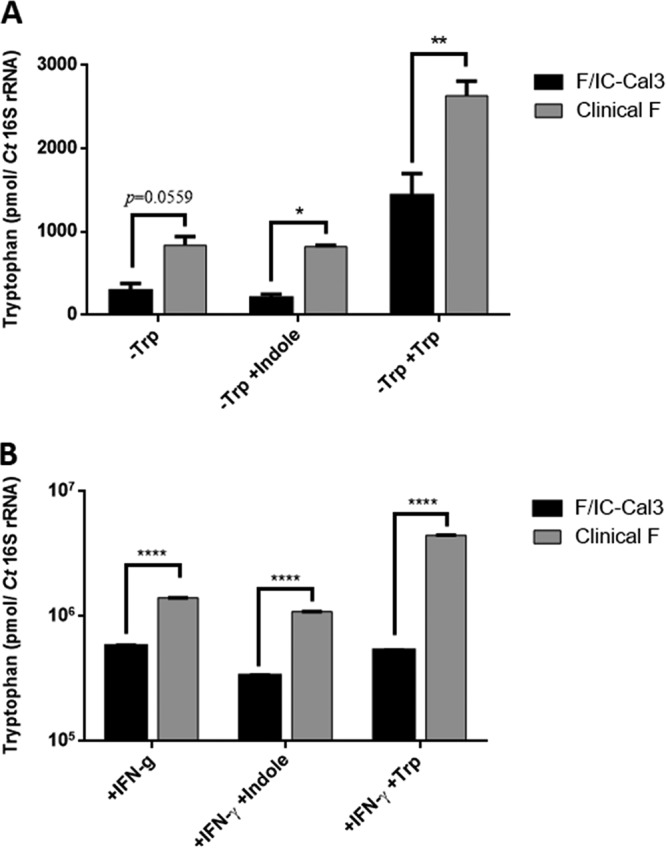
*In vitro* intracellular tryptophan concentrations vary significantly for reference strain F/IC-Cal3 compared with clinical strains F I-IV. (A and B) *Ct*-infected HeLa cells were incubated with either tryptophan-free DMEM medium (−Trp) (A) or complete medium treated with IFN-γ (+IFN-γ) (B) (see Materials and Methods). The medium was supplemented with indole (−Trp +Indole or +IFN-γ +Indole) or tryptophan (−Trp +Trp or +IFN-γ +Trp) as indicated. Infected cells were harvested at 48 hpi, and intracellular tryptophan concentrations were measured using high-pressure liquid chromatography-linked tandem mass spectrometry (see Materials and Methods). Data are presented as the mean tryptophan concentration (in picomoles) normalized to *Ct* 16S rRNA plus SD based on two independent experiments. *, *P < *0.05; **, *P < *0.01; ****, *P < *0.0001.

To determine whether F I-IV can synthesize and utilize tryptophan following tryptophan starvation and indole or tryptophan rescue, intracellular tryptophan concentrations were measured and compared to those of F/IC-Cal3 (Fig. 8A and B). Using IFN-γ to deplete tryptophan, F I-IV had significantly higher intracellular tryptophan concentrations than those of F/IC-Cal3 (*P < *0.0001; Fig. 8B) with a trend for tryptophan-depleted media (*P = *0.0559; Fig. 8A). When tryptophan-depleted cultures were supplemented with indole, intracellular tryptophan concentrations were significantly higher for F I-IV than for F/IC-Cal3 (Fig. 8A, *P < *0.05; Fig. 8B, *P < *0.0001). These findings were supported by the higher levels of intracellular tryptophan concentrations when tryptophan-depleted F I-IV cultures were supplemented with tryptophan compared to F/IC-Cal3 (Fig. 8A, *P < *0.01; Fig. 8B, *P < *0.0001).

## DISCUSSION

As an obligate intracellular bacterium and tryptophan auxotroph, *Ct* relies on host tryptophan pools to replicate. However, despite the fact that *Ct* has a long history of reductive evolution and loss of key functional enzymes (34), the organism has, by and large, retained the tryptophan synthase operon. Previous *in vitro* studies have shown that tryptophan depletion can drive *Ct* into a persistent form, yet with the addition of indole or indole-producing bacteria to the media, urogenital but not ocular strains have been able to synthesize tryptophan and complete their developmental cycle (25, 28).

Comparative genomics of *Ct* strains from different lineages have shown that the ocular strains emerged from the urogenital strains around 10 to 15 million years ago (29). Through subsequent reductive evolution, ocular strains lost their ability to synthesize tryptophan. The low prevalence or lack of indole-producing bacteria in the conjunctiva (35, 36) may have contributed to this loss. However, some indole producers such as Propionibacterium acnes and Escherichia coli have routinely been found in the microbiota of healthy conjunctiva (37). Others have proposed that ocular strains lost their functional operon to maximize fitness (36). During infection and upregulation of IFN-γ, *Ct trpBA* expression is induced, but in the absence of indole, the β subunit catalyzes the deamination of l-serine to produce pyruvate and ammonia (38), an antimicrobial agent that can also induce apoptosis of epithelial cells, especially under higher pH levels (39), similar to those found on the ocular surface of healthy patients (40). Losing tetramer function would prevent the production of this deleterious agent and promote *Ct* growth. Sherchand and Aiyar recently demonstrated that indole derivatives, produced by some gut bacteria and present in serum, can induce the expression of *Ct trpBA*, despite sufficient tryptophan concentrations (41). They found that these indole derivatives act as a derepressor of *Ct* TrpR, and during infection, when indole is absent from the media, the ammonia produced by a functional tryptophan synthase enzyme has bactericidal effects on *Ct* growth. This effect was absent when ocular *Ct* strain A2497 or a urogenital D *trpB*-null strain with a point mutation in TrpB was used (41).

In this study, we identified an F strain that was repeatedly isolated from the endocervix of a woman with asymptomatic *Ct* infections. The F I-IV genomes were 100% identical, suggesting a persistent infection that was able to survive in spite of the host immune response and repeated antibiotic treatment. The strains had a unique α subunit point mutation that caused a frameshift and elongation of the TrpA by two aas unlike any publicly available F strains. This is the first study to document a novel urogenital *Ct* mutation in *trpA* with an *in vivo* advantage for a nonfunctional operon in a clinical setting.

The clinical F TrpA 3D protein model showed that α254N and α255L at the C terminus were not precisely located at the active ligand-binding site of indole. However, the G253D mutation replaced the nonpolar glycine with the acidic, negatively charged aspartic acid near the active α subunit site and the 25-Å-long tunnel that connects both subunits (Fig. 3B; dashed black line). Since the C-terminal residues for the LBCA 5ey5, M. tuberculosis 5tch, and *S.* Typhimurium 1qop are basic, the surface polarity change could alter the structural position and folding of residues in the tunnel (42, 43) with functional consequences for the clinical F tryptophan synthase. Furthermore, while the *Ct* TrpA cannot capture IGP to initiate indole production and does not produce indole, indole is captured by the α subunit, and in other bacterial species, the tunnel has been shown to accommodate indole channeling from the α subunit to the β subunit (44).

The stability of the closed conformation depends on the α subunit movement of α-L6 and the allosteric connection between the α subunit glycine residue (Glyα181) and the β subunit serine residue (Serβ178) (45). Interestingly, none of the *Ct* strains had serine in this position; clinical F and F/IC-Cal3 had alanine, while A/HAR13 had leucine (Fig. 5A). This suggests that *Ct* tetramer function may be somewhat compromised, even for strains with the complete sequence like F/IC-Cal3, and thus inefficient at producing tryptophan. The A/HAR13 TrpA model revealed a truncated protein in the middle of α-L6, explaining the inability of this strain and other truncated strains to form a closed conformational state to capture indole. The allosteric effect between the two subunits is essential to the efficiency of the enzyme and is different among various bacteria. *Ct* TrpB was found to be inactive in the absence of a full-length TrpA (24). In E. coli, while the β subunit is capable of functioning by itself to produce tryptophan from indole, when it is in an αβ configuration or in its physiological complex, the allosteric effect enhances the catalytic efficiency. Surprisingly, in contrast to E. coli, the LBCA β subunit was found to have a fivefold reduction in the catalytic efficiency when it was found in a complex (32). Although the tryptophan synthase enzyme from the LBCA had the highest homology to the enzyme from *Ct*, the allosteric effect between the two subunits was distinct.

Not surprisingly, when clinical F strains were deprived of tryptophan by IFN-γ treatment, they were not able to recover with indole supplementation, similar to ocular strains. When tryptophan was added back to the starved clinical F strains (+IFN-γ –Trp +Trp), recoverable IFUs/ml were significantly higher than the tryptophan-starved clinical strains growing in the presence of indole. F/IC-Cal3 had an overall higher growth rate in comparison to the clinical strains, and the addition of indole or tryptophan yielded significantly higher IFUs/ml. These data are supported by the fact that clinical F strains produced significantly smaller inclusions than F/IC-Cal3 under IFN-γ-induced tryptophan starvation and indole supplementation (Fig. 6). Furthermore, under the same conditions, the clinical F strains contained many aberrant bodies in the inclusions with few mature infectious elementary bodies (EBs) compared to F/IC-Cal3. These collective data indicate the lack of a functional tryptophan synthase for the clinical F strains.

To assess the ability of clinical F strains to regulate *trpRBA* in a transcriptional manner, mRNA expression levels of *trpBA* were measured during tryptophan starvation with subsequent indole and tryptophan rescue. Consistent with previous work using reference *Ct* strains with and without functional tryptophan operons (25, 46), we found that at 24 hpi during tryptophan starvation, F/IC-Cal3 had significantly higher expression levels of *trpBA* compared with the clinical F strains, indicating that the latter strains cannot upregulate *trpBA* expression and therefore are not capable of synthesizing tryptophan efficiently. This conclusion is supported by our findings that clinical F strain *trpBA* expression levels were relatively similar, regardless of treatment conditions, throughout development at 12, 24, 36, 48, and 60 hpi compared to F/IC-Cal3. Transcription of *trpBA* might also be influenced by additional factors. Recent work has shown that the iron-dependent transcription factor, YtgR, can bind to the intergenic region (IGR) between *trpR* and *trpB*, regulating the expression of the operon (47). Although the clinical F IGR and YtgR transcription factor are similar to those of F/IC-Cal3 (not shown), perhaps additional factors influence the operator near or within the αββα tetramer.

Because *Ct* scavenges host tryptophan pools during tryptophan scarcity, intracellular tryptophan concentration decreases due to competition between the organism and host cell (48). We found that when tryptophan was depleted by IFN-γ treatment or tryptophan-depleted media, clinical F strains had higher intracellular tryptophan concentrations compared to F/IC-Cal3, suggesting that F/IC-Cal3 is a stronger competitor at utilizing host tryptophan pools to recover from starvation. When tryptophan-depleted cultures were supplemented with indole, the findings were similar, indicating that F/IC-Cal3 was more efficient at utilizing indole for tryptophan synthesis that in turn was more efficiently metabolized for recovery. These findings were supported by the higher levels of intracellular tryptophan concentrations for the clinical F strains when the cultures were supplemented with tryptophan.

*Ct* is thought to enter a persistent form when tryptophan is depleted by the host immune response, thereby decreasing its metabolic rate to survive (reviewed in reference 49). However, the organism may first scavenge host tryptophan reservoirs to continue its developmental cycle. The risk with the latter scenario is that low tryptophan concentrations in epithelial cells and the surrounding environment may lead to cellular apoptosis (50). Alternatively, entering a persistent state shortly after tryptophan is limited would preserve host cell tryptophan pools as well as provide a protective niche inside the cell for survival. The clinical F strains that we isolated from the same woman resemble the benefits of this *Ct* persistent form during stressful conditions that include repeated antibiotic treatment and nutrient deficiency induced by the host immune response. Resurgence of the infection may have occurred when sufficient tryptophan became available.

*Ct* could have evolved from an ancestor that was initially residing in the gastrointestinal tract (51) where high levels of indole are available. The availability of indole may have also facilitated establishment of the urogenital tract as another tropic site for *Ct* infection. Although the ability to utilize indole is considered an advantage for this obligate intracellular pathogen, perhaps the loss of a functional α subunit through evolution as for the ocular strains has benefited survival. One part of this may be the energy cost savings in not having to synthesize tryptophan. In any case, it appears that we are now starting to see clinical benefit for this loss of function in sexually transmitted strains.

Although mutations in *trpA* that result in a dysfunctional tryptophan operon were thought to be confined only to ocular strains, the discovery of other mutations among F strains in the present study suggests an additional key evolutionary strategy that allows the organism to survive likely via persistence, even when indole is present. Our data indicate that these mutations affected the structure of the αββα tetramer and, thereby, impact the regulation and function of the operon for the synthesis of tryptophan from indole. Furthermore, this study supports the notion that, during IFN-γ-induced IDO activity and depletion of tryptophan, clinical urogenital *Ct* strains can develop persistent infections *in vivo*. These strains may periodically be able to cause an active infection when tryptophan becomes available, perhaps explaining the recurrence of infections over many years for the patient in our study.

## MATERIALS AND METHODS

### Sample collection, C. trachomatis clinical strain isolation, and whole-genome sequencing.

Endocervical flock swabs (Copan, Murrieta, CA) were collected from women attending health clinics in the San Francisco Bay Area as part of a larger study and were supplied deidentified with no trace to the patient’s name. Hence, the institutional review board (IRB) at University of California San Francisco Benioff Children’s Hospital Oakland determined that this research does not involve human subjects.

Swab samples were placed in cryovials containing 1 ml of M4 medium (Thermo-Fisher, Waltham, MA) and kept at −80°C until processed. C. trachomatis (*Ct*) was isolated from a female with four chronological (>4 years) STIs who had been treated with doxycycline or azithromycin for each documented infection. *Ct* was isolated as previously described (52). Briefly, at 48 hpi, infected cells were harvested, propagated, and purified by density gradient centrifugation as previously described (52). Genomic DNA was purified from each isolate, termed F I, F II, F III, and F IV, with subsequent whole-genome sequencing as we described previously (29). *ompA* and *trpRBA* were resequenced for verification as described previously (52). Table S1 in the supplemental material lists the primers used in this study.

10.1128/mBio.01464-19.1 TABLE S1 Primers designed and used in this study as indicated. Download Table S1, DOCX file, 0.01 MB. Copyright © 2019 Somboonna et al. 2019 Somboonna et al. This content is distributed under the terms of the Creative Commons Attribution 4.0 International license.

### Phylogeny of C. trachomatis clinical strains F I-IV *trpRBA* and TrpRBA.

*trpRBA* and TrpRBA sequences from the four isolates F I to IV (F I-IV) were compared for nucleotide (nt) and amino acid (aa) differences using MEGA 7 (https://mafft.cbrc.jp/alignment/software/). Sequences from seven San Francisco Bay Area clinical F strains and 59 F strains from all public databases were aligned with F I-IV and 20 *Ct* reference strains (Fig. 1, Fig. 2). Maximum likelihood trees were constructed as previously described (29) with 10,000 bootstrap replicates. For aa phylogenies, maximum likelihood trees were constructed using Jones-Taylor-Thornton (JTT) substitution model with 500 bootstrap replicates. *Ct* TrpA and TrpB were also aligned with homologs from other organisms using neighbor joining to detect significant subfamily conserved residues that may be evolutionarily related (29).

### Comparative TrpA and TrpB structure analyses of C. trachomatis clinical and reference strains with homologous species.

F I-IV, F/IC-Cal3, and A/HAR13 TrpA and TrpB sequences were uploaded to MODBASE (http://salilab.org/modbase), and comparative protein three-dimensional modeling structures were calculated using MODELLER (53) with pairwise sequence alignment, sequence-sequence, sequence-profile, and PSI-BLAST methods (54). CHIMERA was used for structural visualization (55). Protein structures were analyzed by satisfaction of spatial restraint (56). The reconstructed putative LBCA tryptophan synthase 5ey5 had the highest homology to *Ct* α and β subunits, followed by M. tuberculosis 5tch and *S.* Typhimurium 1qop. *Ct* sequences were aligned with 5ey5, 5tch, and 1qop using Multalign-Viewer extension (57) and Clustal Omega (58). *Ct* TrpA models were then generated based on the 5ey5 protein structure (homology score of 32.16% to F I-IV, 34.43% [based on 183 aa] to A/HAR13 and 32.02% to F/IC-Cal3). *Ct* TrpB models were similarly based on 5ey5 with 56.12% homology to F I-IV and F/IC-Cal3 and 55.87% to A/HAR13.

To authenticate MODELLER structure and functional residue predictions, TrpA domain architectures were determined using Conserved Domain (CD) (59), Protein family (Pfam) (60), Simple Modular Architecture Research Tool (SMART) (61), Transmembrane helix prediction (TMHMM) (62), and SignalP (63) tools. Protein BLAST (64) against the Protein Data Bank (PDB) using a 0.01 cutoff E value and Protein Homology/analogy Recognition Engine (Phyre) (65) were performed to obtain additional homologs that could represent the F I-IV TrpA structure.

*Ct* putative active ligand-binding sites, the indole tunnel, TrpA-TrpB communication (COMM) domain, and nonsynonymous mutations were manually identified and visualized on the predicted models, based on previous work and crystal structures of different Escherichia coli and *S.* Typhimurium mutants (24, 43–45, 66–68).

### C. trachomatis *in vitro* tryptophan starvation and rescue assays.

HeLa229 cell monolayers were inoculated with F I-IV or F/IC-Cal3 at a multiplicity of infection (MOI) of 3. For recoverable infectivity assays, inoculates were incubated in either complete Dulbecco modified Eagle medium (DMEM) (Thermo-Fisher) (–IFN-γ), tryptophan-free DMEM treated with 5 ng/ml human IFN-γ (+IFN-γ) (Sigma-Aldrich), tryptophan-free DMEM with 5 ng/ml human IFN-γ supplemented with 50 μM indole (+IFN-γ –Trp +Indole) (Sigma-Aldrich), or 10 mg/liter tryptophan (+IFN-γ –Trp +Trp). For *trpBA* mRNA transcription assays, inoculates were incubated in either tryptophan-free DMEM (–Trp), tryptophan-free DMEM supplemented with 100 μM indole (–Trp +Indole) or tryptophan-free DMEM supplemented with 10 mg/liter tryptophan (–Trp +Trp). For intracellular tryptophan concentrations, inoculates were incubated in tryptophan-free DMEM, DMEM supplemented with 100 μM indole, DMEM supplemented with 10 mg/liter tryptophan, or with complete CMGH medium supplemented with IFN-γ alone (+IFN-γ), IFN-γ and indole (+IFN-γ +Indole), or IFN-γ and tryptophan (+IFN-γ +Trp). Cultures were incubated for 24 or 48 h (37°C, 5% CO_2_) as indicated.

### Recoverable infectious progeny of C. trachomatis clinical strains F I-IV compared to reference strain F/IC-Cal3 following tryptophan starvation and rescue.

The initial infected cultures were used to infect fresh HeLa cell monolayers, incubated for 44 to 48 h, fixed with methanol, and stained using fluorescein isothiocyanate (FITC)-conjugated *Ct*-specific lipopolysaccharide (LPS) monoclonal antibody (Mab) (Virostat, Westbrook, ME) and Hoechst (to detect nuclei) for enumerating inclusion-forming units per milliliter (IFUs/ml) as we described previously (69).

### Microscopy and transmission electron microscopy.

Morphological characteristics in cell culture were evaluated with fluorescence and transmission electron microscopy (TEM) over the course of *in vitro* development. At 48 hpi, coverslips were fixed and stained, and IFUs/ml were enumerated as described above. Imaris X64 software was used to calculate inclusion area as square micrometers as we described previously (69).

Infected cultures were harvested for TEM characterization as we described previously (69). Briefly, wells were washed with phosphate-buffered saline (PBS) (Mediatech, Herndon, VA) containing 2% glutaraldehyde in 0.1 M sodium cacodylate (Electron Microscope Lab [EML] at University of California [UC] Berkeley), pelleted at 200 × *g*, and prefixed in 2% glutaraldehyde in 0.1 M sodium cacodylate. The samples were refrigerated at 4°C for 24 h prior to postfixation with 1% osmium tetroxide in 0.1 M sodium cacodylate and washed in 0.1 M sodium cacodylate buffer and 0.5% aqueous uranyl acetate overnight. Cells were dehydrated using a graduated ethanol series, embedded in Epon-Araldite resin, and allowed to polymerize. A Riechert Ultracut MT6000 microtome was used to slice 60- to 90-nm sections prior to staining with 2% uranyl acetate and Reynolds lead citrate. A Tecnai 12 TEM (EML) was used for imaging.

### mRNA expression of *trpR* and *trpBA* following tryptophan starvation and rescue.

By using an MOI of 3, 100% of the cells were infected. Infected cells were incubated as described above and harvested at 24 and 48 hpi (69). Briefly, total RNA was extracted and reverse transcribed to cDNA and subjected to quantitative reverse transcription-PCR (qRT-PCR) with two replicates of each cDNA sample and control. 16S rRNA gene expression was used as a control for the number of *Ct* organisms; the human β-actin gene was used as a control for host cell viability. Normalization of the 16S rRNA gene to β-actin gene was used for relative *Ct* growth.

### Intracellular tryptophan concentrations.

At 48 hpi, infected cells were pelleted and resuspended in perchloric acid (Sigma-Aldrich) (10% wt/vol) solution containing 1 mM diethylenetriamine pentaacetate (Sigma-Aldrich) for cell lysis, followed by 4°C centrifugation at 1,500 rpm. Tryptophan concentrations were measured in supernatants using high-performance liquid chromatography-linked tandem mass spectrometry (HPLC-MS/MS) with the EZ-FAAST aa derivatization kit (Phenomenex, Torrance, CA) as previously described (70). Briefly, supernatants containing tryptophan were mixed with an internal standard (tyrosine 3,3-D_2_), and pellets were mixed with a strong cation exchange resin (Phenomenex, Torrance, CA). Derivatization was performed using the manufacturer’s protocols. Derivative products were extracted with isooctane (Sigma-Aldrich), dried under nitrogen, and reconstituted with a mobile phase. Analytes were quantified based on purified standard compounds. Data were normalized against *Ct* 16S rRNA gene transcription levels.

### Statistical analysis.

Data were analyzed using Prism GraphPad V.6. Statistical significance was determined using two-way analysis of variance (ANOVA); *P* values were calculated using Tukey’s multiple-comparison test. A *P* value of <0.05 was considered significant.
