# Supplementary figures and images for: Clinical Persistence of Chlamydia trachomatis Sexually Transmitted Strains Involves Novel Mutations in the Functional αββα Tetramer of the Tryptophan Synthase Operon
Source: mBio. 2019 Jul 16;10(4):e01464-19. doi: 10.1128/mBio.01464-19 (PMC6635532; doi:10.1128/mBio.01464-19)

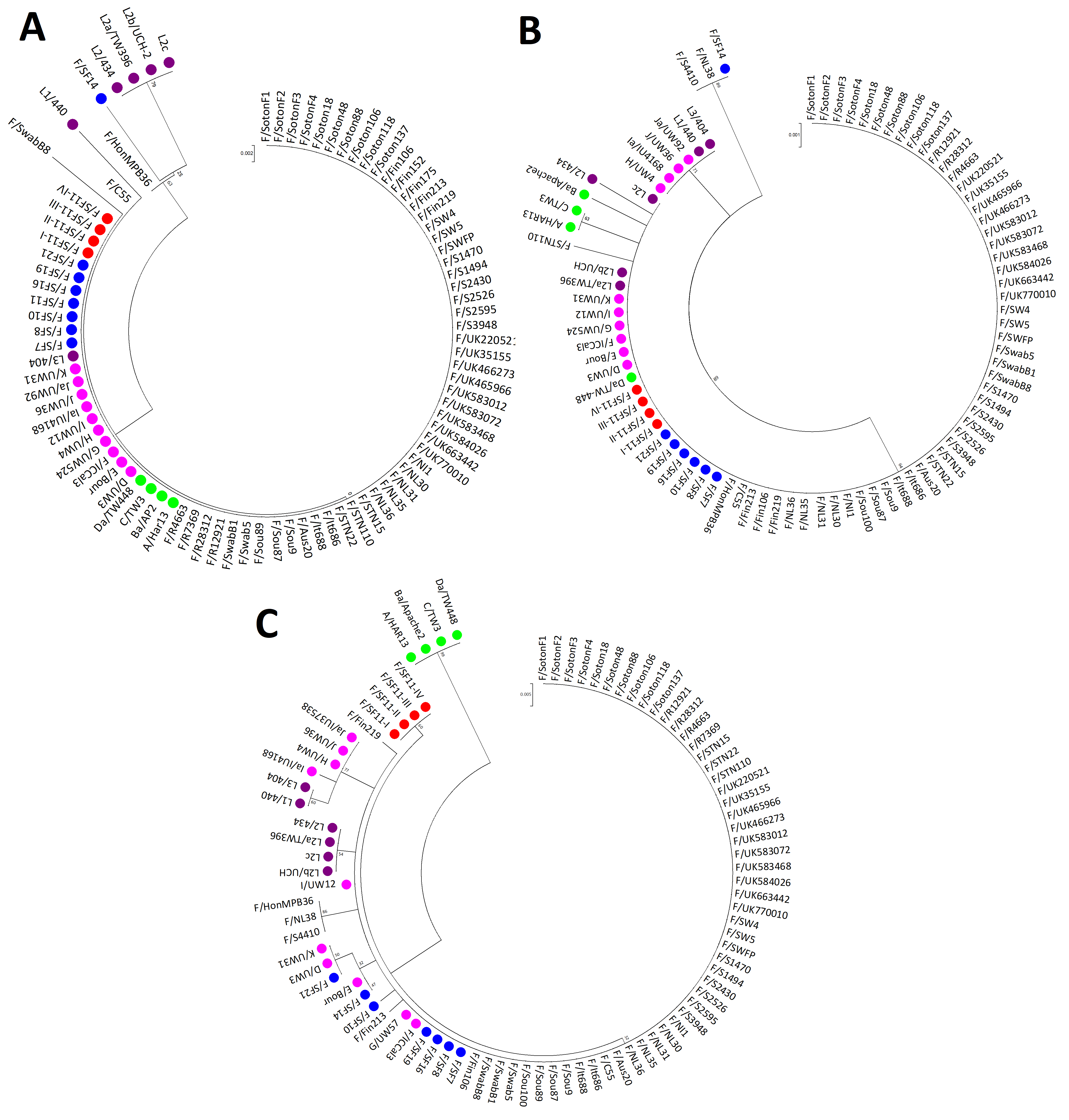

Supplement: FIG S1 [file mBio.01464-19-sf001.tif]
