# Supplementary material for: Clinical Persistence of Chlamydia trachomatis Sexually Transmitted Strains Involves Novel Mutations in the Functional αββα Tetramer of the Tryptophan Synthase Operon
Source: mBio. 2019 Jul 16;10(4):e01464-19. doi: 10.1128/mBio.01464-19 (PMC6635532; doi:10.1128/mBio.01464-19)
Supplement: TABLE S1 [file mBio.01464-19-st001.docx]

**Supplemental Table 1**. Primers designed and used in this study as indicated

| **Gene** | **Primer**  **(PCR & sequencing)** | **Primer sequence (5´→3´)** |
| --- | --- | --- |
| *trpR* | trpR-Forward | TTAGATTTAGCGACGGAAGAAGAG |
|  | trpR-Reverse | AGGCACGCTGTAAGAGGATGA |
| *trpB* | trpB-Forward | AGTATTTGTATGCATGCGTT |
|  | trpB-Reverse | CTGCGGATGATTTGTGGAGAT |
| *trpA* | trpA-Forward | ATTAGCCACCGATGAAGAG |
|  | trpA-Reverse | ATGTTGAATTAGGAGAGTTGTTAT |
| *trpR* | trpR-Fseq | GGAAGAAGAGCGTAGGTTGCGAGCAGATAAGA |
|  | trpR-Rseq | TTTTTGAATATCTTGGAAGCA |
| *trpB* | trpB-Fseq | ACAAATATTCTGTTTTAGCAGTT |
|  | trpB-Rseq | AGATAAGTTAACGATGACGATTTG |
| *trpA* | trpA-Fseq | TGCTTACTAGAAACGAGGGGATTA |
|  | trpA-Rseq | TAATCTATACAGACAACTATGA |
| **Gene** | **Primer**  **(qRT-PCR)** | **Primer sequence (5´→3´)** |
| *16S* | 16SrRNA-9 | GCGAAGGCGCTTTTCTAATTTAT |
|  | 16SrRNA-10 | CCAGGGTATCTAATCCTGTTTGCT |
| *β-actin* | β-actin-3 | GGTGCATCTCTGCCTTACAGATC |
|  | β-actin-4 | ACAGCCTGGATAGCAACGTACAT |
| *trpR* | trpR-FqPCR | AATCAAGAGGAGTCTGGCT |
|  | trpR-RqPCR | CGAATGATATGATAGCGAGAAGC |
| *trpBA* | trpBA-FqPCR | AAATCATCCGCAGAAACAGAGG |
|  | trpBA-RqPCR | ACACCTCCTTGAATCAGAGC |
| *trpB* | trpB-Fclone | CCCCGGTACCATGTTCAAACATAAAC |
|  | trpB-Rclone | CCCCGTCGACTTACTCATAAATTCC |
| *trpA* | trpA-Fclone | CCCCGGTACCATGAGTAAATTAACC |
|  | trpA-Rclone | CCCCGTCGACTTATCCAGGAATAACT |
